# Approach for Elucidating the Molecular Mechanism of Epithelial to Mesenchymal Transition in Fibrosis of Asthmatic Airway Remodeling Focusing on Cl^−^ Channels

**DOI:** 10.3390/ijms25010289

**Published:** 2023-12-25

**Authors:** Susumu Yoshie, Shigeyuki Murono, Akihiro Hazama

**Affiliations:** 1 Department of Cellular and Integrative Physiology, Graduate School of Medicine, Fukushima Medical University, Fukushima 960-1295, Japan; 2 Department of Otolaryngology Head and Neck Surgery, Graduate School of Medicine, Fukushima Medical University, Fukushima 960-1295, Japan

**Keywords:** asthma, airway, fibrosis, epithelial to mesenchymal transition, Cl^−^ channel, cell volume

## Abstract

Airway remodeling caused by asthma is characterized by structural changes of subepithelial fibrosis, goblet cell metaplasia, submucosal gland hyperplasia, smooth muscle cell hyperplasia, and angiogenesis, leading to symptoms such as dyspnea, which cause marked quality of life deterioration. In particular, fibrosis exacerbated by asthma progression is reportedly mediated by epithelial-mesenchymal transition (EMT). It is well known that the molecular mechanism of EMT in fibrosis of asthmatic airway remodeling is closely associated with several signaling pathways, including the TGF-β1/Smad, TGF-β1/non-Smad, and Wnt/β-catenin signaling pathways. However, the molecular mechanism of EMT in fibrosis of asthmatic airway remodeling has not yet been fully clarified. Given that Cl^−^ transport through Cl^−^ channels causes passive water flow and consequent changes in cell volume, these channels may be considered to play a key role in EMT, which is characterized by significant morphological changes. In the present article, we highlight how EMT, which causes fibrosis and carcinogenesis in various tissues, is strongly associated with activation or inactivation of Cl^−^ channels and discuss whether Cl^−^ channels can lead to elucidation of the molecular mechanism of EMT in fibrosis of asthmatic airway remodeling.

## 1. Introduction

Asthma is one of the most common chronic diseases in the world; around 300 million people globally are asthmatic [1]. Patients with asthma experience respiratory symptoms such as wheezing, shortness of breath, chest tightness, and coughing. Asthma is a consequence of the complex interaction of genetic and environmental factors, and its attack and exacerbation are caused by various triggers, such as allergens, cold air, and tobacco [2,3]. The first choice for asthma treatment is the use of inhaled corticosteroids. Low to moderate doses of inhaled corticosteroids can be used to control symptoms in a large number of asthmatic patients. However, for approximately 5–10% of asthmatic patients, even if they inhale the maximum dose of corticosteroids, their symptoms cannot be relieved due to poor steroid responsiveness and/or persistent invasion of inflammatory cells into the airways [4]. The quality of life of these patients has been significantly reduced by the physical burdens of asthma, such as the frequent exacerbations of symptoms and the decrease in respiratory function. They have limited treatment options available, and severe asthma with uncontrolled symptoms leads to death [5]. Therefore, elucidation of the mechanism of asthma is urgently needed.

Asthma is a chronic inflammatory disease that causes airway remodeling, which is characterized by subepithelial fibrosis, goblet cell metaplasia, basement membrane thickening, smooth muscle cell hyperplasia, and angiogenesis [6,7,8,9,10,11,12]. Among them, subepithelial fibrosis worsens as the asthma disease progresses, and epithelial-to-mesenchymal transition (EMT) has been suggested as an important source of fibroblasts that contribute to subepithelial fibrosis [13,14]. This EMT process leads to the migration of an increased number of mesenchymal cells into the subepithelial fibroblast layers, leading to subepithelial fibrosis.

EMT is a phenomenon in which non-motile epithelial cells transdifferentiate into motile mesenchymal cells. Epithelial cells tightly adhere to neighboring cells by forming cell adhesion apparatus such as tight junctions, adherence junctions, and desmosome junctions [15]. On the basal side, epithelial cells are attached to the basement membrane by hemidesmosome junctions. These junctional complexes are critical for maintaining both apical-basal and cytoskeletal polarity within epithelial cells. On the other hand, mesenchymal cells lack apical-basal and cytoskeletal polarity. They exhibit a spindle-like morphology and extend actin-rich membrane projections that facilitate cellular motility. These projections contain sheet-like membrane protrusions called lamellipodia, on the edge of which are spike-like extensions called filopodia [16]. Actin-rich invadopodia cause the degradation of the extracellular matrix, thereby facilitating cell invasion [16,17]. Epithelial cells express cell adhesion molecules such as E-cadherin and ZO-1, while mesenchymal cells lack such expression and exhibit reduced intercellular adhesion. Therefore, during EMT, both polarity and adhesion to surrounding cells and basement membranes are greatly diminished. As a result, they gain enhanced migration and invasion capabilities, leading to their transformation into mesenchymal cells. EMT plays a critical role in diverse in vivo activities, including fibrosis, cancer metastasis, early embryonic development, and tissue repair [18]. A large number of studies have investigated the mechanism of EMT and identified TGF-β1 as an inducer of EMT. When epithelial cells are stimulated with TGF-β1, both the Smad and non-Smad signaling pathways are activated, and consequently, the expression of transcription factors such as SNAIL1, Slug, ZEB1, ZEB2, and TWIST is induced, leading to EMT. In addition, various signaling pathways including the Wnt signaling pathway are also reported to be involved in EMT [19]. However, since the molecular mechanism of EMT has not yet been fully clarified, the detailed elucidation of its molecular mechanism will help to suppress subepithelial fibrosis and ultimately lead to novel therapeutic agents for severe asthma.

The Cl^−^ channels have been reported to play important roles in various physiological phenomena that occur in vivo by transporting Cl^−^. There are various types of Cl^−^ channels that open or close in response to cell membrane potential, intracellular Ca^2+^ concentration, cell volume changes, ligands, and cAMP. Cl^−^ channels are expressed in all types of cells and are widely involved in basic cell functions, such as cell volume regulation [20,21,22], cell migration [23], cell proliferation [22], cell death [22,24], and production [25]. In cell volume regulation, the transport of Cl^−^, K^+^, and Na^+^ via channel, transporter, and/or pump induces passive water flow, leading to cell volume changes such as cell swelling or shrinkage [20,21]. In particular, the volume-sensitive outwardly rectifying Cl^−^ channels (VSOR) are activated after cell swelling caused by hypotonicity. When the cell is swollen by hypotonicity, the extracellular efflux of Cl^−^ through VSOR and that of K^+^ cause the efflux of water molecules from the cell, returning it to its original cell volume [20,21]. It has been reported that Cl^−^ channels that function as VSOR include LRRC8, Ca^2+^-dependent Cl^−^ channels such as some TMEM16 members and tweety homologs (TTYH1, TTYH2, and TTYH3), and voltage-dependent Cl^−^ channels such as ClC-2 and ClC-3. These Cl^−^ channels are deeply involved in the regulation of cell volume. On the other hand, it has also been reported that Cl^−^ channels such as ClC-2, ClC-3, and some TMEM16A members are not associated with a role as VSOR [20,21,22,26,27,28]. Since cell size in each organ and tissue is determined by developmental programs and exhibits a unique cell volume, there is a high possibility that Cl^−^ channels regulate the cell volume and are involved in cell fate decisions during cell differentiation, transdifferentiation, and embryogenesis. In recent years, it has been reported that Cl^−^ channels are involved in cell differentiation [29,30,31], transdifferentiation [32,33], and EMT that causes carcinogenesis and fibrosis [34,35,36]. Thus, EMT caused by the regulation of Cl^−^ channels is thought to be closely related to changes in cell volume. We previously reported that dysfunction of an unspecified number of Cl^−^ channels changes cell volume and promotes EMT in oral squamous cell carcinoma through the activation of the Wnt/β-catenin signaling pathway [34]. Lamouille et al. also reported that cell volume is changed by TGF-β1 during EMT [37]. These reports raise the possibility that Cl^−^ channels and TGF-β1 regulate cell volume and cause EMT. Some studies have reported that EMT, which causes fibrosis, is also closely associated with Cl^−^ channels. Herein, we review recent EMT studies focused on Cl^−^ channels and discuss whether Cl^−^ channels provide clues for elucidating the molecular mechanisms of EMT in the fibrosis of asthmatic airway remodeling.

## 2. The Molecular Mechanism of EMT in Fibrosis of Asthmatic Airway Remodeling

### 2.1. TGF-β Signaling Pathway

TGF-β has been reported to be a key cytokine in the pathogenesis of fibroproliferative diseases of the lungs, kidneys, or livers [38,39,40]. There are three isoforms (TGF-β1, -β2, and -β3) in mammals [41,42], and most studies to date have focused on TGF-β1, which is the most prominent isoform. TGF-β1 is known to be a potent inducer of EMT, leading to fibrosis in tissues such as the airways [43], kidneys [44], and lungs [45]. In asthmatic patients, the expression levels of TGF-β1 are increased in both the airway epithelium and the airway submucosa [46,47]. It has also been reported that eosinophils are a source of TGF-β1 [47,48]. TGF-β1 binds to the constitutively active kinase type II TGF-β receptor, recruits type I TGF-β receptor, and causes the phosphorylation of Smad2/3 [40,43,49,50,51]. The phosphorylated Smad2/3 then translocates to the nucleus to regulate the transcription of target genes, leading to the EMT or airway remodeling. The expression levels of Integrin αvβ6 in epithelial cells have been reported to be increased in response to inflammation stimuli, and activation of TGF-β1 and/or its expression levels are increased [51,52], leading to the EMT. These findings suggest that EMT is caused by a complex interaction between eosinophils, Integrin αvβ6, and TGF-β1. During EMT, epithelial cells acquire the mesenchymal phenotype via downregulation of the expression of epithelial markers such as E-cadherin and up-regulation of the expression of mesenchymal markers such as SNAIL1, which is a well-known master regulator of EMT, as well as cytoskeletal markers such as fibronectin, αSMA, and vimentin, which are essential for enhanced motility [53,54]. On the other hand, it has also been reported that TGF-β1 activates not only the Smad signaling pathway but also the non-Smad signaling pathway to induce EMT. For example, TGF-β1 is known to play an important role in asthmatic airway remodeling by stimulating the PI3K/AKT/GSK-3β signaling pathway. TGF-β1 activates PI3K and AKT, and the activation of AKT phosphorylates GSK-3β, resulting in the inactivation of GSK-3β. Since GSK-3β negatively regulates SNAIL1, inactivation of GSK-3β leads to the activation and nuclear translocation of SNAIL1, as well as the subsequent down-regulation of E-cadherin, leading to the EMT. Yadav et al. reported that the inhibition of aldose reductase prevents TGF-β1-induced EMT in airway epithelial cells and airway remodeling in ovalbumin (OVA)-induced asthmatic model mice via inhibiting the TGF-β1/PI3K/AKT/GSK-3β signaling pathway [55]. Additionally, Liu et al. reported that Lok, which is a traditional folk medicine widely used in northwest China for asthma, inhibits EMT in OVA-induced asthmatic model mice and TGF-β1-induced EMT in airway epithelial cells through inhibiting the PI3K/AKT/HIF-1α signaling pathway [56]. These results indicate that TGF-β1 activates the PI3K/AKT signaling pathway in a Smad-independent manner during EMT, which causes fibrosis in asthmatic airway remodeling. Although TGF-β1 has not been shown to exert an epigenetic gene control mechanism in asthmatic airway remodeling, TGF-β1 causes EMT by inducing the expression of DNA methyltransferases (DNMTs) such as DNMT1, DNMT3A, and DNMT3B in upper airway remodeling caused by chronic rhinosinusitis, indicating that TGF-β1 exerts an epigenetic gene control mechanism. Conversely, the DNMT inhibitor 5-Aza suppresses TGF-β1-induced EMT [57].

### 2.2. Wnt Signaling Pathway

The Wnt/β-catenin signaling pathway has also been reported to contribute to EMT in the fibrosis of asthmatic airway remodeling. Wnt binds to Frizzled receptors, leading to the inhibition of the downstream component GSK-3β. Since GSK-3β negatively regulates β-catenin, inhibition of GSK-3β leads to the cytosolic accumulation of β-catenin and its translocation to the nucleus and subsequent up-regulation of transcriptional factors such as SNAIL1, leading to EMT [13]. It has been reported that high expression levels of Wnt family proteins and β-catenin have been detected in the airways of asthmatic model mice. These elevated expression levels are characterized by airway remodeling, such as subepithelial fibrosis and airway smooth muscle hyperplasia. Suppression of β-catenin expression in the airways of asthmatic model mice attenuated airway remodeling, including subepithelial fibrosis [58]. Furthermore, mesenchymal stem cell (MSC) injection or MSC-derived exosome reduced EMT in the airways of asthmatic model rats through the inhibition of the Wnt/β-catenin signaling pathway [59]. Taken together, these findings demonstrate that the Wnt/β-catenin signaling pathway is highly expressed in asthmatic airways and regulates the development of fibrosis. Furthermore, it has also been reported that the specific gene expression induced by β-catenin depends on the recruitment of the transcriptional co-activator CREB binding protein (CBP) [60]. Moheimani et al. have shown that inhibition of complex formation between β-catenin and CBP due to the use of the small molecule inhibitor ICG-001 results in suppression of EMT in airway epithelial cells [61]. This suggests that activation of β-catenin/CBP complexes contributes to EMT in asthmatic airway epithelial cells.

### 2.3. Other Signaling Pathways

Various signaling pathways other than the TGF-β1 signaling pathway and Wnt signaling pathway have been reported to be associated with EMT in fibrosis of asthmatic airway remodeling. Zou et al. reported that the combination exposure of TGF-β1 and house dust mites induces EMT in airway epithelial cells via activation of the SHH signaling pathway [62]. Feng et al. demonstrated that IL-24 contributes to EMT in asthmatic model mice via the activation of the ERK1/2 and STAT3 signaling pathways and further revealed that IL24-mediated EMT is significantly alleviated by the inhibition of the ERK1/2 and STAT3 signaling pathways [63]. Furthermore, the RhoA/ROCK signaling pathway has also contributed to EMT in OVA-induced asthmatic model mice [64]. Wang et al. reported that inhibition of the crosstalk between the TGF-β1/Smad3 and Jagged1/Notch1 signaling pathways attenuates EMT in OVA-induced asthmatic model mice [65]. These data mean that complex synergistic interactions between the TGF-β1/Smad3 and Jagged1/Notch1 signaling pathways facilitate EMT. Thus, the signaling pathways of EMT that cause fibrosis in asthmatic airway remodeling are diverse and interact with one another to form complex networks. The molecular mechanisms of EMT in fibrosis of asthmatic airway remodeling have not yet been fully elucidated, as new signaling pathways and molecules continue to be identified. There is a high possibility that previously unreported molecules and signaling pathways may contribute to EMT.

## 3. The Roles of Cl^−^ Channels on Morphological Changes Such as Cell Differentiation and Transdifferentiation

Cl^−^ channels have been reported to play important roles in cell volume regulation [20,21,22], cell differentiation [29,30,31], and transdifferentiation [32,33]. In the cell volume regulation mechanism, the transport of Cl^−^, K^+^, and Na^+^ causes a passive flow of water, resulting in changes in cell volume such as cell swelling or shrinkage [20,21]. In particular, it has been reported that VSOR, which is a key player in vertebrate cell volume regulation, is activated by hypotonic stress in order to regulate cellular volume. The extracellular efflux of Cl^−^ through VSOR and that of K^+^ cause the efflux of water molecules from the cell, returning it to its original cell volume [20,21]. Recently, members of the LRRC8 (leucine-rich repeat-containing 8) family have been identified as the central contributors to VSOR [20,21]. Additionally, it has been reported that TTYHs serve as LRRC8-independent VSOR [26,27,28]. Therefore, Cl^−^ channels are considered to be deeply involved in morphological changes, such as cell differentiation and transdifferentiation, that are related to cell volume changes. In previous reports, most studies of cell differentiation and transdifferentiation triggered by regulation of Cl^−^ channels have not been investigated with a focus on cell volume changes; however, those studies have suggested that Cl^−^ channels regulate the specific signaling pathways, the transcriptional factors, and the concentration of intracellular Cl^−^, and that they contribute to control cell differentiation and transdifferentiation in a variety of cells. For example, Hou et al. have reported that ClC-2, which is a voltage-dependent Cl^−^ channel, may function as an important positive regulator in oligodendrocyte precursor cell differentiation through the regulation of various transcriptional factors such as YY1, MRF, Sox10, and Sip1 [66]. Wang H et al. and Wang D et al. have suggested that ClC-3, which is also a voltage-dependent Cl^−^ channel, mediates osteogenic differentiation via the Runx2 pathway [29,67]. Furthermore, Yin et al. reported that ClC-3 plays a role in cell volume regulation as a VSOR and is associated with the fibroblast-to-myofibroblast transition [68]. Chen et al. have shown that the extracellular efflux of Cl^−^ caused by LRRC8 is activated during myogenic differentiation at an early stage, and a moderate amount of intracellular Cl^−^ is necessary for myoblast fusion [69]. It has also been reported that LRRC8 promotes myoblast differentiation by regulating hyperpolarization and intracellular Ca^2+^ signals [70]. These findings indicate that LRRC8 may control cell volume and be closely involved in cell differentiation and transdifferentiation. Additionally, it has been reported that cystic fibrosis transmembrane conductance regulator (CFTR) regulates mesendoderm differentiation from embryonic stem (ES) cells via the β-catenin signaling pathway [31]. CFTR has also been shown to control intestinal lineage differentiation from mouse ES cells [71]. In airway epithelial cells, defective TMEM16A, a Ca^2+^-dependent Cl^−^ channel, promotes differentiation of secretory cells and goblet cells, resulting in goblet cell metaplasia [72,73]. On the other hand, Scudieri et al. suggested that the upregulation of TMEM16A is associated with the differentiation of goblet cells [74]. Furthermore, ClC-2 and the chloride intracellular channel, CLIC4, control the transdifferentiation from fibroblast to myofibroblast via the TGF-β1 signaling pathway [32,33]. These results indicate that Cl^−^ channels are deeply involved in morphological changes such as cell differentiation and transdifferentiation.

## 4. Relationship between Cl^−^ Channels and EMT That Causes Carcinogenesis, Migration, and Invasion on Various Tissues

As mentioned above, Cl^−^ channels have been reported to be closely related to morphological changes such as cell differentiation and transdifferentiation. On the other hand, there are many reports that Cl^−^ channels are also involved in EMT, which is one of the morphological changes and causes carcinogenesis, migration, and invasion on various tissues. For example, the expression levels of CLCA1, which is one of the Ca^2+^-dependent Cl^−^ channels, are significantly lower in colorectal cancer tissues than in normal tissues. Increased expression levels of CLCA1 in colorectal cancer suppress growth and metastasis via inhibition of the Wnt/β-catenin signaling pathway in vitro and in vivo, whereas inhibition of CLCA1 causes the opposite results [75]. These results indicate that CLCA1 controls the EMT process via the Wnt/β-catenin signaling pathway. Xin et al. have reported that the expression levels of CLCA2, which is also a Ca^2+^-dependent Cl^−^ channel, are significantly reduced in cervical cancer cells. Furthermore, the overexpression of CLCA2 inhibits EMT via the inactivation of the p38/JNK/ERK signaling pathway and also inhibits the proliferation, migration, and invasion of cervical cancer cells [35]. Additionally, the expression levels of CLCA2 are significantly lower in nasopharyngeal carcinoma tissues than in noncancerous nasopharyngeal tissues. Overexpression of CLCA2 significantly suppresses EMT through inactivation of the FAK/ERK1/2 signaling pathway. In contrast, knockdown of CLCA2 has the opposite effect [76]. Furthermore, CLCA4 has also been reported to suppress EMT in esophageal cancer, colorectal cancer, liver cancer, and breast cancer by regulating specific signaling pathways such as the PI3K/AKT pathway [77,78,79,80]. These results indicate that the Ca^2+^-dependent Cl^−^ channels CLCA1, CLCA2, and CLCA4 function as tumor suppressors. Recently, TTYHs, which have been reported to act as VSOR, have contributed to EMT, including migration and invasion on cholangiocarcinoma through the Wnt/β-catenin signaling pathway [81]. TMEM16A, a Ca^2+^-dependent Cl^−^ channel other than CLCA and TTYHs, has been associated with cell proliferation, migration, invasion, and tumor growth in various cancers such as glioblastoma [82], breast cancer [83], head and neck cancer [84], and gastric cancer [85]. CFTR, which is a cAMP-dependent Cl^−^ channel, is expressed in various epithelial cells, and CFTR mutations cause cystic fibrosis. CFTR has also been reported to be involved in the EMT of cancer cells [86,87]. Downregulation of CFTR in breast cancer cells enhances malignant phenotypes and is deeply involved in a poor prognosis for breast cancer [87]. Additionally, rather than focusing on a specific Cl^−^ channel, the inhibition of an unspecified number of Cl^−^ channels has been reported to promote EMT in oral squamous cell carcinoma by changing the cell volume and regulating the Wnt/β-catenin signaling pathway [34]. Taken together, the results of these reports indicate that Cl^−^ channels are closely involved in EMT, which causes carcinogenesis, migration, and invasion. Thus, Cl^−^ channels raise the possibility of contributing to EMT, which causes fibrosis in asthmatic airway remodeling.

## 5. Relationship between Cl^−^ Channels and EMT That Causes Fibrosis in the Airways and Other Tissues

In the airways and kidneys, previous studies have reported a tight relationship between Cl^−^ channels and EMT (Table 1). Quaresma et al. found that cystic fibrosis tissues or cells expressing mutant CFTR display several signs of EMT activation, including destructured epithelial proteins, defective cell junctions, increased levels of mesenchymal markers, and EMT-associated transcriptional factors. Furthermore, they suggested that mutant CFTR-triggered EMT is mediated by the transcription factor TWIST1 [88]. Thus, it is possible that temporary dysfunction or downregulation of CFTR may also cause EMT in the fibrosis of asthmatic airway remodeling. Additionally, it has been reported that CFTR expression decreases in unilateral ureteral obstruction (UUO)-induced kidney fibrosis in mice and kidney fibrosis in humans. The downregulation or dysfunction of CFTR in renal epithelial cells is a key event leading to EMT and kidney fibrosis via the aberrant activation of the β-catenin signaling pathway. Conversely, the overexpression of CFTR alleviates fibrotic phenotypes in the UUO model [36]. These results suggest that CFTR dysfunction is a trigger for EMT that causes fibrosis in the airways and kidneys. Furthermore, it has been reported that LRRC8, which functions as VSOR, is involved in EMT in renal tubular epithelial cells derived from fetal kidneys. The inhibition or defectiveness of LRRC8 attenuates TGF-β1-induced EMT phenotypes such as migration [89]. This finding suggests that cell volume changes may actually be linked to EMT, and LRRC8 may also cause EMT in fibrosis of the kidneys and asthmatic airways. Yang et al. have shown that overexpression of the voltage-dependent Cl^−^ channel ClC-5 in the UUO-induced kidney fibrosis mouse model and TGF-β1-treated human renal tubular epithelial cells restores E-cadherin expression, reduces vimentin expression, and inhibits EMT. Conversely, the downregulation of ClC-5 in TGF-β1-treated human renal tubular epithelial cells increases the acetylation of NF-κB and the expression of an invasion-related gene, MMP9, and further potentiates EMT [90]. This suggests that ClC-5 is strongly involved in EMT, which causes kidney fibrosis, through the NF-κB/MMP9 signaling pathway.

Several EMT studies focused on TRP channels and K^+^ channels have been reported (Table 2). Wang et al. and Xu et al. have revealed that TRP channels, which are non-selective cation channels that transmit not only Na^+^ and K^+^, but also Ca^2+^ and Mg^2+^, are associated with EMT in asthma and chronic obstructive pulmonary disease [91,92,93]. TRP channels are widely recognized to respond to temperature, nociceptive stimuli, touch, osmotic pressure, pheromones, and other stimuli from within and outside the cell [94]. In particular, activation of TRPC1 among TRP channels increases intracellular Ca^2+^ concentration, subsequently downregulates the expression of cytokeratin 8 and E-cadherin, and upregulates the expression of αSMA, leading to EMT in airway epithelial cells [91]. Additionally, Pu et al. have shown that TRPC1 promotes EMT in house dust mite (HDM)-induced asthmatic model mice through the activation of the STAT3/NF-κB signaling pathway. It has also been suggested that airway remodeling is alleviated through the suppression of the STAT3/NF-κB signaling pathway in TRPC1^−/−^ mice even after HDM challenge [93]. In addition to TRP channels, KCa3.1, a calcium-dependent K^+^ channel, has been proposed as a new target for fibrosis of the airways and lungs. The expression levels of KCa3.1 are increased in the airway epithelium of asthmatic patients compared with those of healthy people, and the KCa3.1 current is larger in asthmatic airway epithelial cells compared with healthy airway epithelial cells. Several features of TGF-β1-induced EMT have been reported to be suppressed by selective blockers of KCa3.1 [95]. These findings indicate that various anion and cation channels control the flow of their respective ions and are involved in EMT through the activation or inactivation of specific signaling pathways.

These reports indicate that various Cl^−^ channels, including LRRC8, CFTR, and ClC-5, may be closely involved in EMT that causes fibrosis in asthmatic airway remodeling.

## 6. Conclusions and Future Directions

Asthmatic airways are characterized by airway remodeling such as subepithelial fibrosis, goblet cell metaplasia, basement membrane thickening, angiogenesis, and smooth muscle cell hyperplasia. In particular, elucidation of the mechanism of EMT that causes fibrosis is urgently needed, since exacerbation of asthma is linked to fibrosis. The molecular mechanism of EMT in the fibrosis of asthmatic airway remodeling has been reported to be caused by diverse signaling pathways, including the TGF-β1 signaling pathway and the Wnt signaling pathway. However, the EMT mechanism is driven by complex interactions with various molecules and signaling pathways, and the molecular mechanism of EMT has not yet been fully clarified.

**Figure 1:**
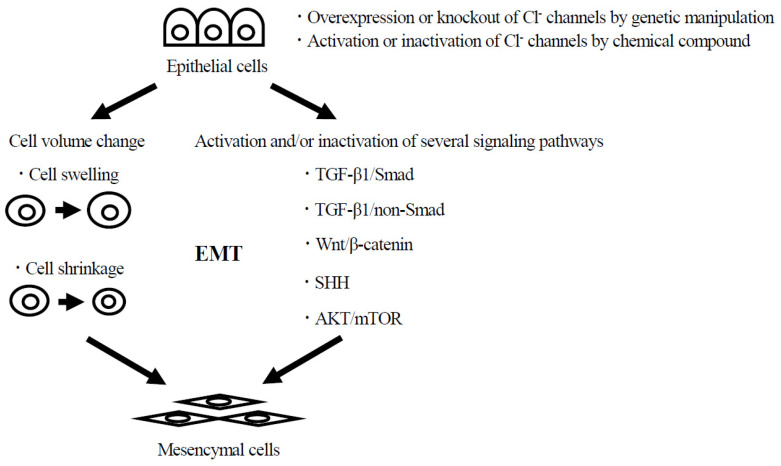
Elucidation of the EMT mechanism in fibrosis of asthmatic airway remodeling by Cl^−^ channel. Activation or inactivation of Cl^−^ channels by genetic manipulation or chemical compound changes the flow of Cl^−^ and water molecule, leading to cell swelling or cell shrinkage. Activation or inactivation of Cl^−^ channels by genetic manipulation or chemical compound also activates or inactivates specific signal pathways such as the TGF-β1/Smad signaling pathway, TGF-β1/non-Smad signaling pathway, Wnt/β-catenin signaling pathway, SHH signaling pathway, and AKT/mTOR signaling pathway. Consequently, Cl^−^ channels may contribute to EMT in fibrosis of asthmatic airway remodeling.

Cl^−^ channels have been reported to play an important role in cell volume regulation [20,21,22]. In the cell volume regulation mechanism, the transport of Cl^−^, K^+^, and Na^+^ causes a passive flow of water, resulting in changes in cell volume such as cell swelling or shrinkage [20,21]. In particular, it has been reported that VSOR plays a key role in vertebrate cell volume regulation. In short, Cl^−^ transport mediated by those Cl^−^ channels causes a passive flow of water, resulting in changes in cell volume. Since cell size in each organ and tissue is determined by developmental programs and exhibits a unique cell volume, there is a high possibility that Cl^−^ channels regulate the cell volume and are deeply involved in cell fate decisions such as cell differentiation, transdifferentiation, and EMT. In fact, there have been many reports that Cl^−^ channels are closely associated with cell differentiation, transdifferentiation, and EMT, which causes carcinogenesis in various tissues. Additionally, Cl^−^ channels such as CFTR and ClC-5 have been shown to be strongly involved in EMT leading to kidney fibrosis; therefore, there is a high possibility that Cl^−^ channels are involved in the fibrosis of asthmatic airway remodeling. In the near future, it is expected that Cl^−^ channels will provide a new clue for elucidating the mechanism of EMT that causes fibrosis in asthmatic airway remodeling and may become a new target for suppressing fibrosis in patients with severe asthma. However, although it has been suggested that Cl^−^ channels control EMT through the activation or inactivation of specific signal pathways such as the Wnt/β-catenin signaling pathway, there are only a limited number of EMT studies focused on cell volume changes. We [34] and Lamouille et al. [37]. have suggested that the change in cell volume is associated with EMT. Furthermore, LRRC8, which functions as VSOR, is involved in EMT in renal tubular epithelial cells. On the other hand, in hearts, myocardial necrosis is caused after ischemia/reperfusion-induced myocardial infarction. Uramoto et al. showed that the activation of endogenous CFTR channels in myocardial cells suppresses myocardial necrosis [96]. This finding suggests that chloride ions are released from myocardial cells via activated CFTR and that the cell swelling caused by ischemia/reperfusion-induced myocardial infarction is inhibited, thereby providing protection against necrotic myocardial injury. These reports indicate that Cl^−^ channels control cell volume and are involved in several phenomena. Thus, elucidating the EMT mechanism from the perspective of cell volume changes with a focus on Cl^−^ channels may also provide a new clue for elucidating fibrosis in the airways of patients with severe asthma (Figure 1). Investigating the direction of Cl^−^ transport via Cl^−^ channels using patch clamps or Cl^−^-sensitive fluorescent dyes before and after EMT stimulation will be the first step in clarifying the relationship between EMT and cell volume changes associated with Cl^−^ transport. Furthermore, during the EMT process, monitoring cell size and differentiation status in real time will help clarify the relationship between cell volume and EMT. In addition, intentional increases or decreases in cell volume caused by hypo- or hyper-osmolarity conditions may promote or suppress EMT. Strict control of cell volume in some way has the potential to control not only EMT but also various morphological changes, including cell differentiation and transdifferentiation. If a relationship between EMT that causes fibrosis in asthmatic airway remodeling and cell volume regulation via Cl^−^ channels is revealed, cell volume regulation via Cl^−^ channels will lead to a new treatment for fibrosis in asthmatic airways.

## Figures and Tables

**Table 1:** Reports on fibrosis focusing on Cl^−^ channels and EMT.

Author (Year)	Channel	Reference Number
Zhang et al. (2017)	CFTR (cAMP-dependent Cl^−^ channel)	[36]
Quaresma et al. (2020)	CFTR (cAMP-dependent Cl^−^ channel)	[88]
Yang et al. (2019)	ClC-5 (voltage-dependent Cl^−^ channel)	[90]

**Table 2:** Reports on fibrosis focusing on ion channels other than Cl^−^ channels and EMT.

Author (Year)	Channel	Reference Number
Pu et al. (2007)	TRPC1 (non-selective cation channel)	[93]
Arthur et al. (2015)	KCa3.1 (calcium-dependent K^+^ channel)	[95]

## Data Availability

Not applicable.
